# SALaMA study protocol: a mixed methods study to explore mental health and psychosocial support for conflict-affected youth in Detroit, Michigan

**DOI:** 10.1186/s12889-020-8155-5

**Published:** 2020-01-10

**Authors:** Lindsay Stark, Mackenzie V. Robinson, Ilana Seff, Wafa Hassan, Carine Allaf

**Affiliations:** 1 Brown School, Washington University in St. Louis, Campus Box 1196, One Brookings Drive, St. Louis, MO 63130 USA; 2 Mailman School of Public Health, Columbia University, 722 West 168th St., New York, NY 10032 USA; 3 Global Educational Excellence, 2455 S. Industrial Hwy, Ann Arbor, MI 48104 USA; 4 Qatar Foundation International, 1225 New York Avenue NW, Suite 500, Washington, DC, 20005 USA

**Keywords:** Mental health, Psychosocial support, Youth, Acculturation, Education, Refugees, Immigrants, Middle East, MENA, Immigrant paradox

## Abstract

**Background:**

Families resettling to the U.S. from conflict-affected countries in the Middle East and North Africa (MENA) face countless challenges. These families must cope with experiences of armed conflict and forced migration while also assimilating to a new society. According to the ‘immigrant paradox,’ time spent in a new country can compound the effects of migration and assimilation challenges and lead to deteriorated mental health. This study aims to assess the psychosocial wellbeing of MENA-born or first-generation adolescents attending school in the Detroit metropolitan area (DMA) to understand how schools, families, and communities play a role in supporting these adolescents’ wellbeing.

**Methods:**

The quantitative component of this mixed methods study will involve a self-administered survey with a sample of students whose responses will be linked to academic records and behavioral assessments. The survey will utilize validated instruments to measure depressive and anxiety symptoms (Hopkins Symptom Checklist-37A), hope (Children’s Hope Scale), resilience (Child and Youth Resilience Measure-12), externalizing and prosocial behavior (Hopkins Symptom Checklist-37A, Strengths and Difficulties Questionnaire), school belonging (Psychological Sense of School Membership), and peer relationships (Multidimensional Scale of Perceived Social Support). Differences in outcomes will be analyzed across two strata: students born in the MENA region and first-generation students whose parents immigrated to the US from the MENA region. The qualitative component will involve semi-structured key informant interviews with parents, school administrators, educators, and mental health providers, and focus group discussions (FGDs) with a purposive sample of adolescents born – or whose parents were born - in the MENA region. The FGDs will include a participatory ranking activity where participants will be asked to free-list and rank ideas about how schools can better support students like them. Thematic content analysis will be conducted to identify common themes.

**Discussion:**

This study will contribute evidence about the wellbeing of adolescents who come from – or whose parents come from - conflict-affected countries currently living in the U.S. Findings can be used to inform program and policy development to enable schools and their community partners to serve this population more effectively.

## Background

Adolescence is a critical phase of human development, during which physical, neural, and psychological growth are readily influenced by external factors. Experiences during adolescence can have a profound effect on health and wellbeing that last through adulthood [1]. While this developmental period can be challenging for any individual, adolescents who have been – or whose parents have been - resettled from the Middle East and North Africa (MENA) region face a number of unique challenges that may increase their risk of adverse mental and psychosocial health outcomes. Following escape from political conflict and violence at home, forced migrants often endure a multitude of hardships throughout their flight, from a lack of basic resources, to detention, humiliation, and physical and sexual abuse [2]. Those who resettle in the United States may not only struggle to process their loss of home and experiences of migration, but must also adapt to a new language and culture, and learn to navigate complicated legal and public service systems [3]. Further, adolescents coming from conflict-affected settings often spend months or years out-of-school prior to resettlement and may struggle to catch up with their peers academically, all the while at risk of facing routine discrimination, micro-aggressions, and bullying in their new communities [4, 5].

Arab immigrant, refugee, and U.S.-born Arab-American adults report higher levels of poor mental health outcomes than non-Arab Americans, including depression and anxiety [6, 7]. However, despite the emerging literature on adults from Arab-majority countries, fewer studies have focused on adolescents within this same population. The few studies that have been undertaken have shown that children and adolescents resettling in high-income countries, such as the U.S., tend to be at elevated risk of poor psychological outcomes, such as mood and anxiety disorders, compared to those living in the country of origin [3, 8]. Given the known adversities stemming from conflict, migration and acculturation stressors, these findings may not come as a surprise.

What is perhaps more surprising is that time spent in a resettlement country has been shown to exacerbate these mental health risks, both among immigrant adults and adolescents [9]. Within this ‘immigrant paradox,’ prolonged time lived in the U.S. can result in deteriorated mental health and wellbeing among immigrant children and adolescents compared to first generation U.S.-born children of immigrants [6, 10, 11]. While this finding may seem counterintuitive given the considerable acculturative stress experienced by this population, it has been hypothesized that those newer to the country may fare better relative to those who have resided for longer periods of time due to protective factors unique to immigrant populations, including living in ethnic enclaves upon first arrival to the U.S. Known as the ‘ethnic density effect,’ this phenomenon has been theorized to contribute to better physical and mental health outcomes among immigrants living in high income countries compared to those living in other areas [12–17].

The available evidence points to several related protective factors for adolescents from conflict-affected countries who have resettled in high-income countries. Perceived community acceptance and support is a critical factor influencing psychosocial health outcomes among this population, together with parental support, family connectedness, and continued links with the cultural and religious practices of the origin country, among others [18, 19]. The erosion of such protective factors coinciding with increases in ‘acculturative stress’ can lead to a decline in overall mental health of immigrant adolescents over time [10, 11].

Schools in particular are thought to play a central protective role for youth from these settings. For example, studies in the U.S. have found that refugees and asylum-seekers reporting a strong sense of school belonging (or high school social capital) tended to have lower levels of psychosocial distress [20–22]. In addition to educating students who have been resettled, and connecting them to a broader community of peers, schools can also provide an environment in which families may build new support networks and relationships. For example, the Colorado Department of Human Services recently found that school support contributed not only to students’ integration, but to the integration of parents as well, with parents becoming markedly more involved in their children’s schools over time [23]. Practitioners and researchers alike have become increasingly interested in the role of schools in promoting healthy development and improved psychosocial wellbeing among resettled refugees and asylum-seekers, and nascent evidence points to the potential of such school-based interventions in high-income countries.

Within an increasingly hostile political climate, a broader evidence base is urgently needed to develop programs and policies that enable schools and their partners to serve this population effectively. Ongoing conflict in countries such as Syria, Iraq, Libya, and Yemen, contribute to the large number of refugees and asylum-seekers in the U.S. Syria and Iraq, for example, were two of the top five origin countries of refugees and asylum-seekers resettled in the U.S. in 2016 and 2017, together representing over a quarter of new arrivals in each of those years [24]. Additionally, given the recent influx of Arabic newcomers nationally, it is important to explore the nuanced differences in experiences and psychosocial adjustment among adolescent students, with the understanding that needs may differ depending on where and how long they have resided in the US. To bridge these gaps in evidence and practice, the SALaMA study aims to assess the mental health and psychosocial wellbeing of adolescent students from conflict-affected, Arab-majority countries living in the largest Arab ethnic enclave in the U.S., with special attention paid to whether they were born in the U.S or abroad.

## Methods/design

### Study setting

This mixed methods, cross-sectional study will take place across three public charter high schools located in the Detroit Metropolitan Area (DMA) of Michigan, which has an extensive history of economic and migratory fluctuations [25, 26]. Around 15.6% of the DMA’s 4.3 million people live below the federal poverty level with an average life expectancy that is 5 years shorter than comparable populations in other major US cities, including New York City, San Francisco, and Dallas [27, 28].

This setting was chosen for the present study due to its relatively high percentage of Arab immigrant, refugee, and asylum-seeking students. The DMA has the second largest number of Arabs and Arab-Americans in the U.S., coming second only to Los Angeles [29]. Historically, this area has been a popular destination for MENA immigrants and forced migrants/refugees, with 46% of Dearborn’s population identifying as Arab [30, 31]. Cohorts of immigrants and forced migrants/refugees from the MENA region have continually arrived in the U.S. through what are now referred to as the “three waves of Middle East migration,” though the exact time periods that define each wave can vary [30, 32]. The first wave arrived in the latter half of the nineteenth century, and was comprised of individuals who largely sighted seeking opportunities for educational and financial success as the primary reason for their migration [33]. The second wave arrived post-World War II when the U.S. received a large influx of MENA immigrant and refugee families fleeing conflict and poverty, primarily from Lebanon, Iraq, and Yemen [17, 30, 33]. The majority of these families settled in the DMA mostly due to the abundance of jobs available through the Ford Motor company. This second wave is generally credited as fostering the community’s social cohesion and Arab-American identity [33–36]. The third wave comprises those who emigrated primarily from Syria, Palestine, Jordan, Egypt, and the Arabian Peninsula after the U.S. Immigration Act was enacted in 1965 [17, 30]. This wave is considered to have had the largest number of MENA migrants, especially with the onset of the Lebanese Civil War in 1975, the Gulf War of 1991, and subsequent invasions of Iraq and Syria post-September 11th [17, 32]. Migrants in this wave tended to join their families who had migrated in previous waves, leading to a sevenfold increase in the number of MENA migrants to the U.S. [17]. Most of these migrants settled in the DMA, creating one of the largest current Arab ethnic enclaves outside of the MENA region [17, 30, 34, 36].

### Study design, sample and recruitment

The three participating schools in our study have an estimated combined enrollment of 2000 students, with the majority of students teachers and staff identifying as Arab.

The primary research questions (RQs) are:

- RQ 1: What are the current levels and experiences of mental and psychosocial wellbeing among students who have migrated from abroad, including those of Arab origin, and how do they compare to first generation Arab-American students in the selected schools?
- RQ 2: What elements in the family, school, community, and district contribute to the mental and psychosocial wellbeing of these students in the selected schools?
- RQ 3: What kinds of additional support do adolescents and adults identify to improve the mental and psychosocial wellbeing of these students in the selected schools?

This study will utilize a mixed methods design. The quantitative portion of the study will involve a self-administered survey with students enrolled in Arabic language classes aged 13+ years from participating schools. The qualitative portion of the study will involve (1) semi-structured key informant interviews with parents, school administrators, educators, and mental health providers and (2) focus group discussions (FGDs) with a purposive sample of adolescents who were born – or whose parents were born - in the MENA region. The FGDs will include a participatory ranking activity in which participants will be asked to free-list and rank ideas about how high schools can better support resettled students from conflict-affected countries. The research protocol has been approved by the Institutional Review Board (IRB) at Washington University in St. Louis (IRB- 201905151) and the participating schools’ principals.

### Quantitative data collection

A structured survey will be self-administered by respondents using school computers. Data will be collected on students’ socio-demographics, public services usage, experiences prior to and following resettlement (if applicable), and psychosocial wellbeing. Due to the unique ethnic/racial composition of students at these high schools, researchers will utilize census sampling to ensure adequate sample sizes for each stratum (further described below). The research team will visit every Arabic class at each school to explain the study and distribute parental consent forms. Aside from students aged 18+ years, only students whose parents sign and return consent forms will be approached for assent on the day of data collection. Student assent will be obtained prior to the survey and students will be informed of their rights as participants. The survey was developed in English and translated into Arabic; both versions will be available to all students who participate. The research team will also work with the school offices to access records of student performance, such as academic grades and behavioral assessments. Several steps will be taken to protect participant privacy throughout data collection and analysis. The survey will be self-administered using a password-protected school computer. Completed surveys will automatically upload to a secure server that is accessible only by research staff and the local files will be erased from the device. Data files will be de-identified and indexed by a participant code assigned as part of the study to reduce the possibility that confidential information will be leaked beyond the intended research staff in the event that study instruments are lost or stolen. All data will be disposed of 7 years after completion of the study and all electronic records will be permanently deleted. All data will be disposed of 7 years after completion of the study and all electronic records will be permanently deleted.

The survey will examine the following key outcomes using scales/instruments previously validated to measure various dimensions of mental health and psychosocial wellbeing with refugee and asylum-seeking populations in high-income countries: depressive and anxiety symptoms, hope, resilience, externalizing and prosocial behavior, school belonging, and peer relationships (See Table 1).

**Table 1:** Key psychosocial outcomes to be measured

Instrument	Rationale/Outcome Measured	Components Used	Values
Children’s Hope Scale [37]	Measures students’ hope, as related to their agency and pathways for meeting goals	6-item scale	Average score on all six questions that may take a value from 1 to 6, with a higher score reflecting greater hope
Strengths and Difficulties Questionnaire (SDQ) [38]	Used to screen for poor dimensions of mental health	Two of the sub-scales from the original five: Peer Problems and Prosocial sub-scales	Each sub-scale may take value from 0 to 10, with a higher score reflecting greater peer problems relationships or more pro-social behaviors, respectively
Child and Youth Resilience Measure (CYRM) [39, 40]	Measures resilience among children and adolescents while allowing for cultural variation across multiple settings, and has been previously validated with adolescents in refugee/asylum-seeking contexts	12-item version of the original 28-item scale	May take a value from 12 to 36, with a higher value reflecting greater resilience
Hopkins Symptom Checklist [41]	Measures multiple dimensions of mental health, including depressive symptoms, symptoms of anxiety, and externalizing symptoms; has been previously validated with refugee/asylum-seeking adolescents in multiple languages	37-item version of the original 58-item checklist	All sub-scales may take a value from 1 to 4, with a higher score reflecting greater symptomology
Stressful Life Events checklist [42]	Measures exposure to eleven stressful lifetime events and experiences pertaining to drastic changes in the family in the last year	12-item scale	May take a value from 0 to 12, with a higher value reflecting exposure to a greater number of events
Multidimensional Scale of Perceived Social Support (MSPSS) [43, 44]	Measures perceived social support from three respective sources: friends, family, and a significant other; has been validated with multiple adolescent populations, including Arab American adolescents	12-item scale	May take a value from 0 to 7, with a higher value reflecting greater perceived social support
Psychological Sense of School Membership (PSSM) scale [20, 45]	Measures students’ sense of belonging in their school environment; has been used and validated with adolescent refugee/asylum-seeking populations in the U.S.	18-item scale	May take a value from 18 to 90, with a higher value reflecting a greater sense of school belonging

### Qualitative data collection

#### Focus group discussions

The researchers will conduct FGDs with students to understand the risk and resilience factors associated with post-migration experiences of students from conflict-affected, Arab-majority countries. With assistance from school staff, purposive sampling will be used to prioritize the inclusion of students. Inclusion criteria include 1) being 13 years of age or older, 2) being born in -- or having a parent born in – a MENA country 3) active school enrollment status, and 4) fluency in English or Arabic. It is anticipated that up to 12 FGDs, stratified by gender, will be required to reach saturation. FGDs will be conducted using a semi-structured guide consisting of several open-ended questions, including a series of probing questions, to develop an understanding of the students’ social environment at school. Questions include:

- *What are some of the challenges of making new friends at your school?*
- *How do you get along with other students that do not speak Arabic or come from Arab countries?*
- *Who are some of the adults in the school that you would go to if you had a personal problem, like a problem at home or with another student?*

The FGD will then lead into the Participative Ranking Methodology [46], a qualitative research method in which student participants will be asked to discuss and prioritize ways in which high schools can support students resettled from conflict-affected countries.

#### Parent/caregiver interviews

Up to 30 in-person parent interviews will be conducted across the three schools until saturation is reached. Inclusion criteria for the purposive selection of parents includes 1) having a child who is first-generation or who was born in a MENA country and 2) fluency in English or Arabic. A semi-structured interview guide will be used to explore parental relationships with their children, as well as pre- and post-migration experiences for families. The interview guide is designed to gain insight into the challenges and opportunities for strengthening students’ supportive environments. Sample questions include:

- *How do you feel about your new community?*
- *What are some of the conversations you have had in your family about mental health?*
- *What are some challenges you’ve had as a family since being here?*

#### Key informant interviews

Approximately 10–20 KIIs will be completed with representation from a diverse range of stakeholders who work with resettled families from Arab-majority countries. Separate semi-structured interview guides were developed for use with educators, service providers, administrators, and government officials, respectively. Similar to the parent/caregiver interviews, these guides are meant to gain insight into the challenges and opportunities for strengthening students’ supportive schools and communities.

FGDs and interviews with parents/caregivers and school staff will take place in a private room provided by schools. Key informant interviews will take place at a location of the participant’s choice. Informed consent will be obtained from all FGD and interview participants and consent forms will be available in both English and Arabic. All FGDS and interviews will be recorded (contingent on participant consent) and transcribed. Arabic interpreters will be available for any participants who require such assistance. Researchers will take extensive field notes during qualitative data collection. Handwritten notes will be typed into a digital format and de-identified immediately after an interview or group discussion. The handwritten notes will then be destroyed. Typed notes and audio recordings will be stored on a password-protected computer, with only research staff allowed access to the data. Any transcripts produced from audio files of qualitative data collection sessions will be de-identified and stored in the same manner as the audio files and field notes. All data will be disposed of 7 years after completion of the study. All paper records will be shredded prior to disposal and audio files will be deleted.

### Quantitative sample size calculations and analysis

The survey sample will consist of two study strata including (1) students who were born outside of the US, and (2) students who were born in the US but whose parents immigrated to the US. The necessary sample size for the quantitative sample has been calculated to detect a difference in the proportion of students with depression, at *d* = 0.40, between the two study strata. Calculations were conducted assuming α < 0.05, 80% power, and a one-to-one ratio of students in both sample groups. As a result, a total of 196 students (98 in each stratum) are required.

Differences in the outcomes of interest between the two strata will be tested using a first-order linear regression; tests will be deemed significant at *p* < 0.05. Multivariate linear regressions will then be employed to assess whether bivariate relationships between group membership and the outcomes of interest hold when controlling for other covariates, including various sociodemographic characteristics, measures of academic performance, and stressful life events. Lastly, effect modification will be assessed by examining whether any covariates deemed to be associated with the outcomes of interest in the previous step have a differential impact on the outcomes across the two strata. The significance of potential effect modifiers will be identified through the use of interacted linear regressions, where group membership is interacted with key covariates. Additional analyses will explore similar relationships between group membership and secondary outcomes around academic performance. All quantitative analyses will be conducted using Stata14 [47].

### Qualitative assessment and analytic plan

Qualitative data will be analyzed using a combination of inductive and deductive thematic analysis, allowing themes to emerge from the data inductively while using themes from previous studies with similar population [48, 49]. Additionally, because data will be analyzed from each stratum separately, constant comparative method will be used to compare and contrast themes across strata [50].

## Discussion

The SALaMA study is the first study to explore the mental health and wellbeing of adolescents who come from – or who have parents who came from – the MENA region and settled in the US. While the challenges associated with migrating to a new country may influence the degree to which newcomer students are able to adapt to their new country, it is also important to understand how this adaptation process might differ for students whose families resettle within more insulated Arab enclaves. The unique ethno-cultural makeup of this study’s setting will allow the findings to speak to the ethnic density effect and its correlation with mental health and psychosocial wellbeing among MENA adolescents residing in an ethnic enclave. The findings can also help to explore if and how the immigrant paradox pertains to this unique population, building on previous studies [51, 52]. All findings will be shared directly with participating schools and organizations as well as through publication in peer-reviewed journals.

There remains a dearth of evidence-based solutions for developing more inclusive policy and program development for refugees and forced migrants. We hope our findings will be used to inform interventions at the district, state, and national levels in high income countries to enable schools and their community partners to serve this population more effectively.

## Data Availability

The de-identified datasets generated and/or analysed during the current study may be available from the corresponding author on reasonable request.

## Abbreviations

DMA: Detroit Metropolitan Area
FGDs: Focus Group Discussions
MENA: Middle East and North Africa region
